# Surgical treatment and long-term outcomes of low-grade myofibroblastic sarcoma: a single-center case series of 15 patients

**DOI:** 10.1186/s12957-021-02454-5

**Published:** 2021-12-07

**Authors:** Jong-Ho Kim, Woosuk Choi, Hwan Seong Cho, Kyu Sang Lee, Joseph Kyu-hyung Park, Baek-Kyu Kim

**Affiliations:** 1 Department of Plastic and Reconstructive Surgery, Seoul National University College of Medicine, Seoul National University Bundang Hospital, 82 Gumi-ro 173beon-gil, Bundang-gu, Seongnam, 463-707 Korea; 2 Department of Orthopedic Surgery, Seoul National University College of Medicine, Seoul National University Bundang Hospital, Seoul, Korea; 3 Department of Pathology, Seoul National University College of Medicine, Seoul National University Bundang Hospital, Seoul, Korea

## Abstract

**Background:**

Low-grade myofibroblastic sarcoma (LGMS) is a poorly studied, rare, soft tissue sarcoma. LGMS is characterized by a low malignancy potential, tendency for local recurrence, and low likelihood of distant metastases. However, no studies have reported on the surgical treatment method and its long-term outcomes.

**Methods:**

We included all patients treated for LGMS at our institution between March 2010 and March 2021. Medical charts were retrospectively reviewed to collect demographic information, as well as information about the clinical course, tumor characteristics, and outcomes. Statistical analysis was performed to identify the factors associated with the recurrence rate.

**Results:**

Fifteen patients who underwent surgical treatment were enrolled in this study. There were seven cases in the upper extremities, four in the trunk area, three in the lower extremities, and one in the head and neck area. There were no metastatic cases and two cases of local recurrence.

**Conclusions:**

The incidence of LGMS in the extremities or trunk may be higher than expected based on the current literature. Univariate analysis showed that local tissue invasion and surgical method could be associated with local recurrence. Although further large studies are needed to establish risk factors of local recurrence or extent of resection margins, based on our study, wide local excision under the proper diagnosis is the most important treatment.

## Introduction

Low-grade myofibroblastic sarcoma (LGMS) is an extremely rare type of malignant tumor that was first reported by Mentzel et al. [1] These tumors originate from the mesenchyme and show myofibroblastic proliferation with fibromatosis-like features [2]. LGMS was first classified as a new group of soft tissue and bone tumors by the World Health Organization in 2002, and this classification was maintained in 2013 [3]. LGMS is characterized by a low malignancy potential, tendency for local recurrence, and low likelihood of distant metastases [1, 4]. LGMS has been reported to occur in deep soft tissues of the head and neck region, extremities, trunk, and abdominal cavities [5, 6]. However, LGMS has also been reported in other locations such as the posterior chest wall [7], breasts [2], vulva [8], and fingers [9]. Only a few clinical studies of this disease have been conducted so far, and there is still no consensus on the optimal surgical method or standard treatment [10]. To the best of our knowledge, no previous case series has reported surgical methods and their long-term outcomes. Here, we report surgical methods used to treat patients with LGMS and their long-term outcomes to inform clinical treatment of LGMS.

## Methods

We investigated all patients surgically treated for LGMS at our institution between March 2010 and March 2021. This study was approved by the Ethics Committee of Seoul National University Bundang Hospital. All clinical information, including sex, age, location, size, and follow-up period, was retrospectively collected. Surgical methods, histopathological findings, and treatment outcomes were retrospectively reviewed (Table 1).

**Table 1:** Patient characteristics and outcome data

Patient no.	Sex/age	Location	Primary or referred	Tumor size (cm)	Local tissue invasion	Metastasis	Surgical method (resection margin)	Recurrence	f/u period (months)	FNCLCC grade
**1**	F/57	Lt. medial elbow	Primary	2.9 × 2.4 × 2.0	(−)	(−)	WLE (2 cm)	(−)	25	1
**2**	M/27	Rt. 3rd finger proximal phalanx	Primary	2.2 × 2.0 × 1.0	(+, bone)	(−)	En-bloc excision Limb salvage surgery	(+; local recurrence)	71	1
**3**	F/45	Lt. paramedian anterior chest wall	Primary	4.6 × 4.0 × 3.5	(+, muscle)	(−)	En-bloc excision	(+; local recurrence)	30	1
**4**	M/68	Rt. deltoid	Referred	2.9 × 1.2 × 1.1	(+, muscle)	(−)	WLE (3 cm)	(−)	18	1
**5**	M/50	Lt. shoulder	Referred	0.7 × 0.5 × 0.4	(−)	(−)	WLE (3 cm)	(−)	25	1
**6**	F/45	Rt. forearm	Referred	(−: no residual mass)	(−)	(−)	WLE (3 cm)	(−)	18	2
**7**	F/20	Lt. shoulder	Primary	2.8 × 2.4 × 2.0	(−)	(−)	WLE (3 cm)	(−)	24	2
**8**	M/68	Rt. shoulder	Referred	2.3 × 2.1 × 1.2	(+, muscle)	(−)	WLE (3 cm)	(−)	60	2
**9**	F/36	Lt. lateral thigh	Referred	1.7 × 1.4 × 0.8	(−)	(−)	WLE (3 cm)	(−)	15	1
**10**	M/62	Rt. inguinal	Primary	5.9 × 5.8 × 4.9	(−)	(−)	WLE (3 cm)	(−)	10	1
**11**	M/61	Rt. forearm	Primary	3.7 × 3.3 × 2.8	(−)	(−)	WLE (2 cm)	(−)	38	1
**12**	M/49	Lt. temple	Referred	2.7 × 2.1 × 0.6	(−)	(−)	WLE (2 cm)	(−)	20	1
**13**	M/58	Lt. hand 2nd webspace	Primary	3.9 × 3.7 × 2.6	(−)	(−)	En-bloc excision	(−)	18	1
**14**	F/70	Rt. distal thigh	Referred	2.3 × 2.0 × 0.5	(−)	(−)	WLE (3 cm)	(−)	15	2
**15**	F/46	Rt. forearm	Referred	1.2 × 0.5 × 1.1	(−)	(−)	WLE (2 cm)	(−)	20	2

*Rt.* right, *Lt.* left, *WLE* wide local excision, *FNCLCC* Federation Nationale des Centres de Lutte le Cancer system

### Surgical method

The primary tumor was widely excised to achieve a negative surgical margin. In all cases, negative margins were confirmed using intraoperative frozen biopsy. The routine resection margin was 3 cm but adjusted according to the location of the tumor or the surrounding structures. The defect site was reconstructed by primary closure, local flap, skin graft, or free flap, based on the defect size and tumor location.

### Histopathological analysis

Diagnosis was based on the histopathological features. Each case was analyzed using routine hematoxylin and eosin staining. For further characterization of the lesions, immunohistochemical studies were performed for all cases using the following stains: smooth muscle actin, desmin, beta-catenin, and Ki67. In cases referred from another hospital, the histopathological results performed at the other hospital were confirmed preoperatively at the Pathology Department of our institution. Tumor grade was determined using the Federation Nationale des Centres de Lutte le Cancer system.

### Statistical analysis

Overall, local recurrence-free survival was calculated using the Kaplan-Meier method. Univariate analysis was performed by log-rank analysis to assess the factors associated with recurrence rate. Differences were considered significant at *p* < 0.05. A multivariate analysis using the Cox proportional hazards regression model was not performed due to the small sample size. SPSS (version 22.0; SPSS Inc., Chicago, IL, USA) was used to perform all statistical analyses.

## Results

Fifteen patients who were surgically treated and documented at our center were included. The study group comprised eight men and seven women with an average age at diagnosis of 50.8 years (range, 20–71 years). The follow-up period ranged from 10 to 61 months (mean, 27.1 months). The primary tumors were located in the upper extremity (*n* = 7), trunk (*n* = 4), lower extremity (*n* = 3), and head and neck (*n* = 1). Seven patients were primary cases and eight patients were referred cases who underwent primary simple excision in another hospital. The average largest diameter was 3.1 cm (range, 0.7–5.9 cm). Wide excision was performed in 13 patients, and en-bloc excision including surrounding normal tissue was performed in two patients. Negative margins were confirmed intraoperatively in the resection margins of all patients. There were four cases of local tissue invasion, including three cases of muscle and one case of bone. The average resection margin was 2.54 cm. There were no metastatic cases and two cases of local recurrence occurred at 8 and 12 months, respectively. Chemotherapy and radiotherapy were not administered to any patient. In the log-rank analysis, local recurrence was associated with the surgical method and local tissue invasion, which were significant (*p* = 0.002 and *p* = 0.014, respectively). However, a multivariate analysis was not performed due to the small sample size. The two cases of recurrence are summarized below.

### Case 1

**Fig. 1:**
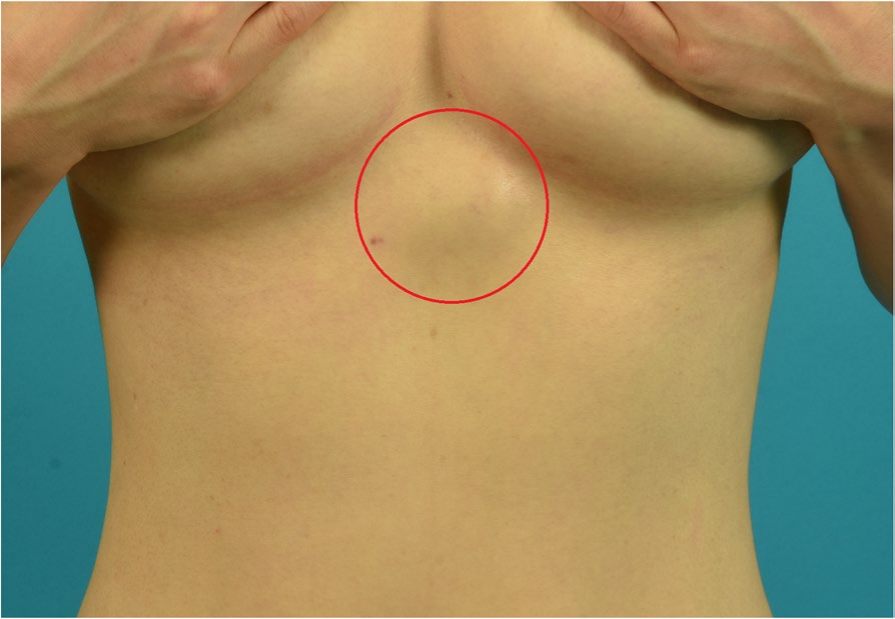
Physical examination revealed a tender mass in upper abdomen, approximately 4 × 4 cm in size

**Fig. 2:**
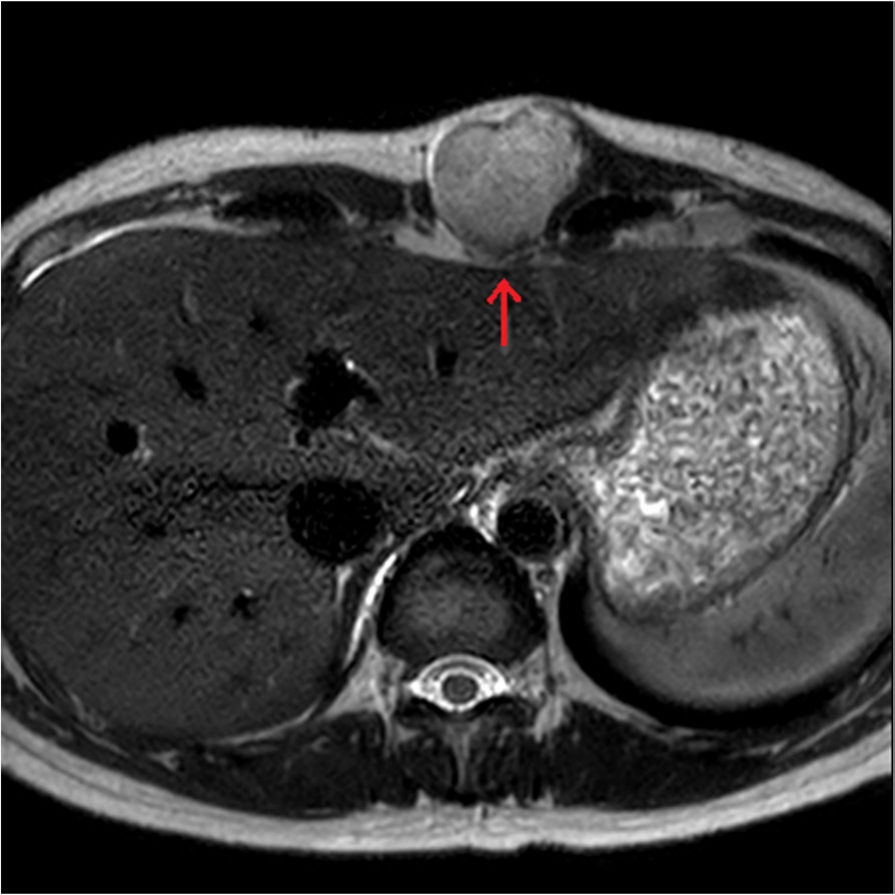
MRI showed a lobulated mass (4.1 × 3.8 × 4.8 cm) in the left paramedian anterior chest wall. MRI revealed the invasion of rectus abdominis muscle (red arrow)

A 45-year-old female patient presented to our department with a complaint of an enlarging lesion in her upper abdomen. Physical examination revealed a tender mass, approximately 4 × 4 cm in size (Fig. 1). Under suspicion of lymphoma following abdominal computed tomography, a core needle biopsy was performed, and a spindle cell proliferative lesion was identified. Chest wall magnetic resonance imaging (MRI) showed a lobulated mass (4.1 × 3.8 × 4.8 cm) in the left paramedian anterior chest wall (Fig. 2). The lesion was resected, including as much of the surrounding tissues as much as possible. However, it was not possible to obtain a sufficient resection margin due to the location of the lesion. Tumor recurrence was suspected 8 months after the surgery due to the presence of a palpable mass, and therefore, an excision was performed. The recurrent lesion was resected with clear margins, and no further recurrence was observed 22 months postoperatively.

### Case 2

**Fig. 3:**
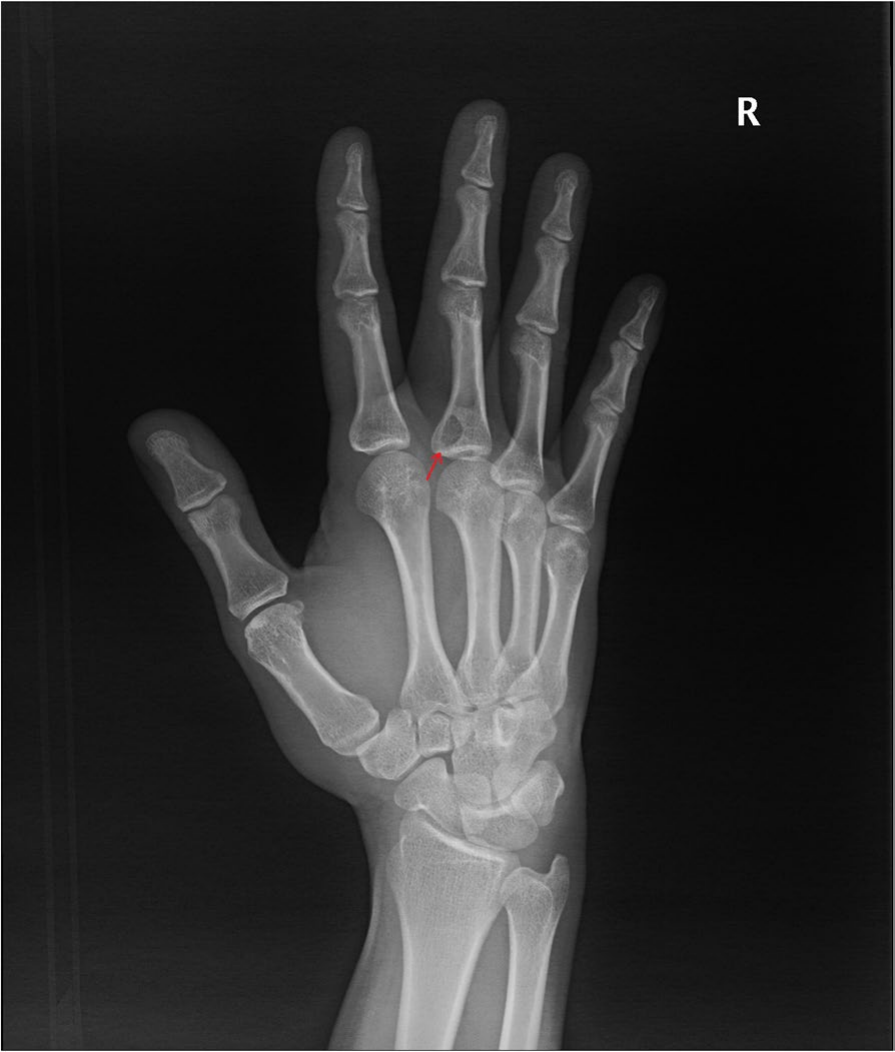
X-ray revealed a bony defect of right third proximal phalanx (red arrow)

**Fig. 4:**
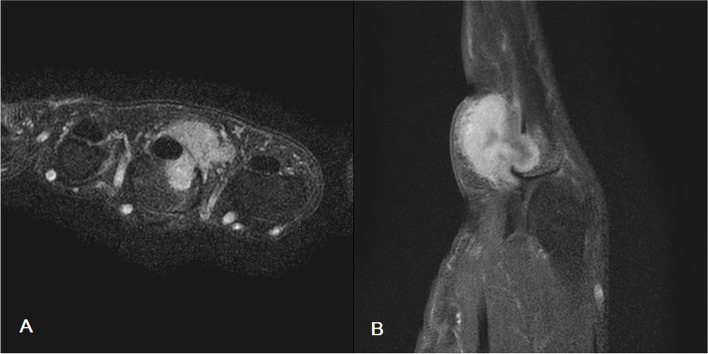
MRI of the finger confirmed the presence of a mass (1.9 × 1.8 × 1.9 cm) encircling the third flexor digitorum tendon, and bony invasion was observed. **A** Axial view (T2-weighted image). **B** Sagittal view (T1-weighted, fat suppressed image)

**Fig. 5:**
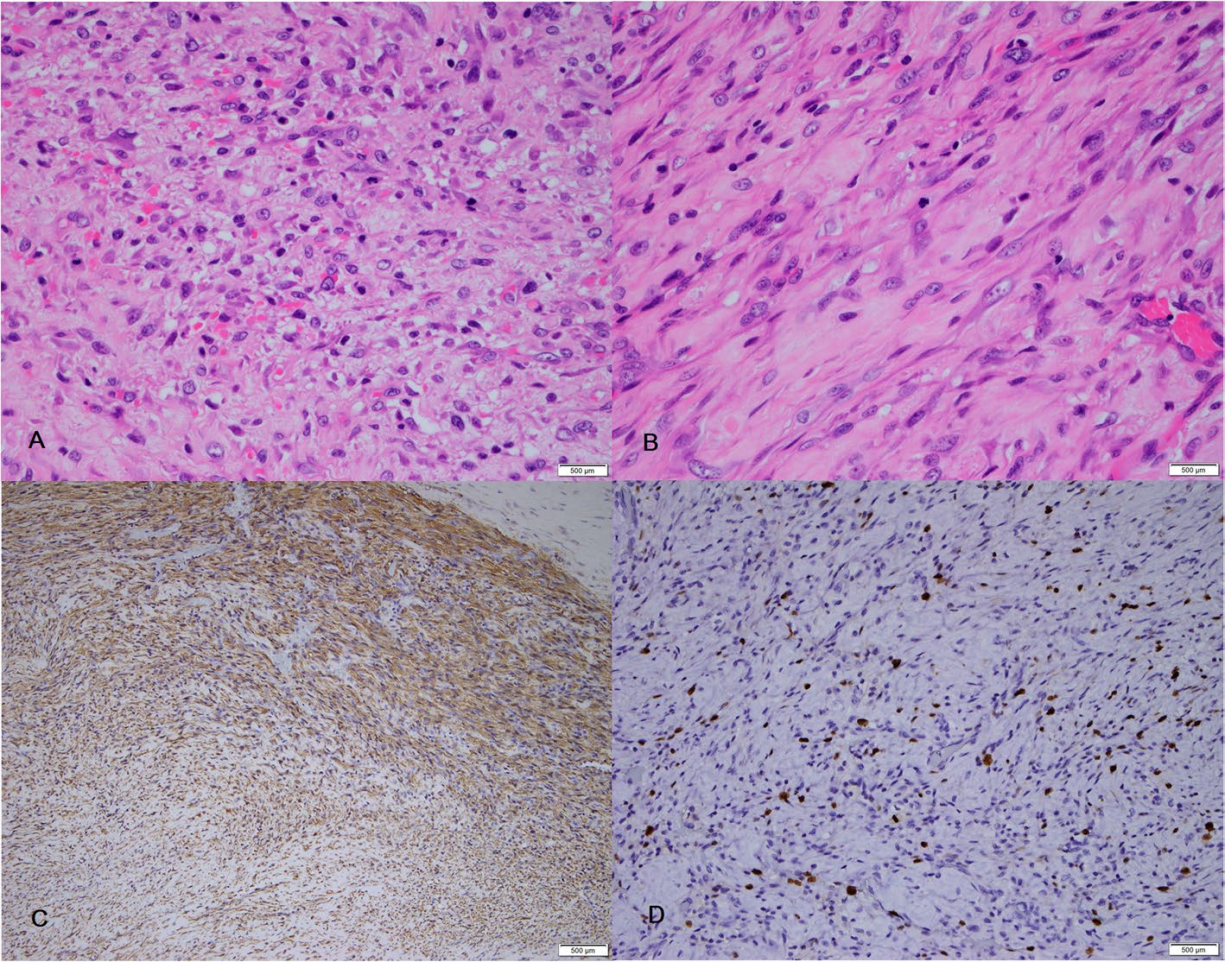
Histopathologic finding. A Hematoxylin and eosin staining (× 40) showing hypercellular areas with a proliferation of spindle cells. **B** Hematoxylin and Eosin staining (x100) showing interlacing fascicles of spindle shaped tumor cells. **C** Immunohistochemistry showing positive staining for smooth muscle actin. **D** Positivity for Ki-67 staining was noted in more than 10% of the tumor cells

A 27-year-old male patient was admitted to our department with a 1.5-cm painless mass on the right third metacarpal joint that occurred 3 months previously. X-ray revealed bond defect of right third proximal phalanx (Fig. 3). MRI of the finger confirmed the presence of a mass (1.9 × 1.8 × 1.9 cm) encircling the third flexor digitorum tendon, and bony invasion was observed (Fig. 4). LGMS was confirmed by an incisional biopsy (Fig. 5). The lesion, including the surrounding tissue, was excised intraoperatively and the neighboring nerves and vessels were saved. The phalangeal bone was reconstructed using an iliac bone graft. Twelve months after surgery, a locally palpable mass was identified, and re-excision was performed with clear margins. No further recurrence was observed 59 months postoperatively.

## Discussion

LGMS is an extremely rare type of tumor that occurs primarily in adult patients with a slight male predominance. The rarity of this type of tumor makes it difficult to draw definitive conclusions regarding clinical characteristics, prognostic factors, and appropriate treatment. The present study is one of the largest series based on data from a single institute.

### Clinical characteristics and prognostic factors for local recurrence

The two largest clinical studies of LGMS were those of Mentzel et al. (18 patients) and Montgomery et al. (15 patients) [1, 11]. LGMS most commonly occurs in the head and neck region with the most common site of occurrence being the oral cavity, especially the tongue [6]. Contrary to previous case reports and case series that reported the oral cavity as one of the most common sites of occurrence in the head and neck region, there were no such cases in our study. Although there is a possibility of selection bias due to the retrospective nature of our selection process, this suggests the possibility that the incidence of LGMS in the extremities or trunk is higher than previously reported. A recent population-based study in the USA reported 49 cases of LGMS with a 5-year overall survival rate of 71.6% [12]. In this population-based study, contrary to previous reports, LGMS occurred most commonly in the extremities (40.8%), followed by the head and neck region (26.5%). In our study, the extremities were the most common sites (66.6%), followed by the trunk (26.7%), and head and neck (6.7%). In our study, a multivariate analysis was not performed due to the small number of patients; however, univariate analysis showed the possibility that local tissue invasion and surgical method were associated with local recurrence. The reason why the local recurrence rate was lower in our study than in previous studies is that sufficient resection margins could be achieved due to the location of the tumors in the extremities and trunk. In the previous reports, tumors were located in the head and neck, including the oral cavity, where it is difficult to obtain sufficient resection margins [5, 13]. Some studies have reported that one-third of LGMS cases in the head and neck have a higher risk of local recurrence and metastasis than cases in other regions, which supports the results of our study [5].

### Standard treatment of LGMS

Due to the rarity of LGMS, its optimal treatment remains unclear. In two previous studies reporting 18 and 15 patients with LGMS, only four and three patients, respectively, received chemotherapy or radiation [1, 11]. The roles of radiotherapy and chemotherapy in LGMS remain unclear and there is no guideline to apply these treatments [12]. In some case reports, adjuvant chemotherapy showed significant clinical improvement in the presence of metastases, which was associated with prolonged progression-free survival [7]. The current study of Xu Y et al. investigated the role of chemotherapy and radiotherapy in treating LGMS and included 96 patients from the Surveillance, Epidemiology, and End Results databas e[14]. They concluded that chemotherapy and radiation played limited roles in the outcomes of LGMS and should not be included in the standard treatment. Although successful control of LGMS was possible without chemotherapy or radiotherapy in all our cases, the treatment options depend on the location of tumor which is easily excision or not.

Resection margins are generally accepted as predictive factors for local recurrence; however, the width of resection margins remains controversial, even for other soft tissue sarcomas [15]. In our study, negative margins were confirmed in all patients, and no chemotherapy or radiation therapy was administered. In all cases with resection margins ≥ 2 cm, no recurrence was observed during the follow-up period. There were only two cases of local recurrence without metastasis, and they were successfully controlled by local re-excision during the follow-up period. Two local recurrences occurred in patients who underwent en-bloc excision because wide local excision was difficult due to the location of the tumor (in either the paramedian chest wall or the proximal phalanx). Although resection margins were free of the tumor, it has been reported that a closer margin from the tumor may increase the risk of local recurrence [16]. Some studies have reported that a resection margin ≤2 mm was considered positive [17]. It is not yet clear whether these surgical factors influence tumor recurrence, but it is assumed that wide excision is important as a treatment for LGMS. Given these data, wide local excision, including surrounding normal tissue, seems to be the primary treatment and may become a standard treatment for management of LGMS. Although further studies are still needed to establish the optimal width or extent of resection margins, based on our study, wide local excision under the proper diagnosis is the most important treatment.

This study had several limitations. The sample size was too small for statistical analysis, and prospective studies using a larger number of patients are required. Second, as mentioned above, there is a possibility of selection bias due to the retrospective nature of the study. A large number of prospective studies are needed to properly understand the prognosis and treatment of LGMS.

## Conclusion

In this study, we reported the surgical methods used to treat LGMS and the long-term outcomes of this treatment. The incidence of LGMS in the extremities or trunk may be higher in our study than has been previously reported. Factors associated with local recurrence include local tissue invasion and surgical method. We propose that wide local excision with a sufficient resection margin is important for the treatment of LGMS.

## Data Availability

Data has been anonymized and is kept with the authors. It is available upon request.
